# Salvianolic Acid A Protects against Acetaminophen-Induced Hepatotoxicity via Regulation of the miR-485-3p/SIRT1 Pathway

**DOI:** 10.3390/antiox12040870

**Published:** 2023-04-03

**Authors:** Fan Tang, Zhecheng Wang, Junjun Zhou, Jihong Yao

**Affiliations:** Department of Pharmacology, Dalian Medical University, Dalian 116044, China; tangfansyxy@163.com (F.T.); wangzc03@dmu.edu.cn (Z.W.)

**Keywords:** salvianolic acid A, APAP, miR-485-3p, SIRT1, oxidative stress, inflammation

## Abstract

The vast majority of drug-induced liver injury is mainly attributed to acetaminophen (APAP) overdose. Salvianolic acid A (Sal A), a powerful water-soluble compound obtained from *Salvia miltiorrhiza*, has been confirmed to exert hepatoprotective effects. However, the beneficial effects and the exact mechanisms of Sal A on APAP-induced hepatotoxicity remain unclear. In this study, APAP-induced liver injury with or without Sal A treatment was examined in vitro and in vivo. The results showed that Sal A could alleviate oxidative stress and inflammation by regulating Sirtuin 1 (SIRT1). Furthermore, miR-485-3p could target SIRT1 after APAP hepatotoxicity and was regulated by Sal A. Importantly, inhibiting miR-485-3p had a hepatoprotective effect similar to that of Sal A on APAP-exposed AML12 cells. These findings suggest that regulating the miR-485-3p/SIRT1 pathway can alleviate oxidative stress and inflammation induced by APAP in the context of Sal A treatment.

## 1. Introduction

Acetaminophen (APAP) is widely used as an analgesic and antipyretic drug [1]. It is considered to be safe when administered at therapeutic doses, but in cases of APAP overdose, acute poisonings can occur. One recent study has reported that toxic levels of APAP may increase the risk of neurodegeneration and development of Parkinson’s disease (PD) [2]. Moreover, the intake of large doses of APAP results in severe liver injury and even acute liver failure [3].

Nontoxic doses (up to 4 g per day in adult humans) of APAP are eliminated by glucuronide and sulfate conjugation. The initial step in APAP hepatotoxicity is the formation of the reactive metabolite N-acetyl-p-benzoquinoneimine (NAPQI) by cytochrome P450 2e1 (CYP2E1) [4]. After an overdose, the reactive intermediate NAPQI forms mitochondrial protein adducts, which cause oxidative stress within the organelle and subsequently initiate signaling cascades that result in programmed necrosis [5]. Then, necrotic cells lead to the release of cellular proinflammatory mediators and the recruitment of inflammatory cells such as neutrophils, monocytes and lymphocytes, which are thought to be cytotoxic [6]. However, recently, as shown in experimental models and humans, the deleterious or beneficial roles of the inflammation induced by APAP in liver regeneration have been controversial [7].

Sirtuin 1 (SIRT1) has attracted much attention recently due to its protective effect against oxidative stress and inflammation [8,9]. A recent study showed that SIRT1 could control reactive oxygen species (ROS) and inflammation through the IL-1β/nuclear factor-kappa B (NF-κB) pathway in an APAP model [4]. Therefore, targeting SIRT1 contributes to the prevention of APAP-induced liver injury.

In a recent study, microRNAs (miRNAs) effectively modulated SIRT1 expression [10]. MiRNAs, which are endogenous ~22-nt RNAs, can cleave or post-translationally repress target mRNAs [11]. Because miRNAs may serve as important regulators of SIRT1 expression, we hypothesize that the miRNA-SIRT1 axis may be involved in oxidative stress and inflammation in different disease processes.

Salvianolic acid A (Sal A), which is extracted from Danshen, exhibits various biological activities, such as antioxidant activity and anti-inflammatory and antiapoptotic effects [12,13]. Our previous studies indicated that Sal A inhibited apoptosis and inflammation in acute liver injury induced by concanavalin A [14]. However, whether Sal A could protect against liver injury induced by APAP and the underlying molecular mechanisms need to be investigated.

The objectives of the current study were as follows: (1) to explore Sal A and its potential to reduce liver damage caused by APAP; (2) to elucidate whether SIRT1 was associated with Sal A-mediated protection against APAP-induced hepatotoxicity; and (3) to test whether Sal A regulates the expression of SIRT1 via a specific miRNA.

## 2. Materials and Methods

### 2.1. Chemicals

Sal A (≥98%) and APAP were obtained from Winherb (Shanghai, China) and Sigma-Aldrich (St. Louis, MO, USA), respectively. Alanine/aspartate aminotransferase (ALT/AST) and glutathione (GSH) assay kits were purchased from Jiancheng Corp. (Nanjing, China). Western blot analysis was performed with antibodies specific for manganese superoxide dismutase (MnSOD), NF-κB (Proteintech Group, Wuhan, China), p66Shc (BD Biosciences, San Jose, CA, USA), SIRT1 (Abcam Ltd., Cambridge, UK) and β-actin (ZSGB-BIO, Beijing, China).

### 2.2. Animals and Treatments

C57BL/6 mice (8 weeks) were randomly divided into five groups: control; control+Sal A (30 mg/kg/day); APAP; APAP+Sal A (15 mg/kg/day) and APAP+Sal A (30 mg/kg/day). The mice were pretreated orally with Sal A for 3 days. On the last day, 1 h after orally administering Sal A, the mice received a single intragastric injection of APAP (300 mg/kg) [15]. After 4 h, plasma and liver tissue were collected. All of the procedures were performed in compliance with the Guide for the Care and Use of Laboratory Animals.

### 2.3. Analysis of Serum ALT/AST Activities

Serum ALT and AST levels were assessed according to the manufacturer’s instructions.

### 2.4. Analysis of Liver GSH/CAT/H_2_O_2_/MDA Levels

GSH, catalase (CAT), hydrogen peroxide (H_2_O_2_), and malondialdehyde (MDA) levels in livers were tested using commercial enzymatic kits according to the manufacturer’s instructions.

### 2.5. Liver Histological Observation

After fixation and embedding, liver samples were cut into 5 µm sections and stained with hematoxylin and eosin (H&E).

### 2.6. Cell Culture

AML-12 (alpha mouse liver 12) cell line was established from hepatocytes from a mouse (CD1 strain, line MT42) transgenic for human TGF alpha, which has often been used for APAP-induced liver injury [16,17]. AML-12 cells were purchased from the American Type Culture Collection (ATCC, Rockville, MD, USA) and cultured according to a published protocol [18].

### 2.7. Cell Viability Assay

Cell Counting Kit-8 (Biomake, Houston, TX, USA) was used for the cell viability assay. After being treated, the cells were incubated with CCK-8 for 2 h and then measured at 450 nm.

### 2.8. Immunofluorescence Analysis of SIRT1

The cells were treated as previously described in 24-well plates. After being fixed and permeabilized, the cells were blocked in BSA. Then, the cells were incubated with an antibody specific for SIRT1 (Proteintech Group, Wuhan, China) and exposed to a goat anti-rabbit Cy3 polyclonal antibody. Hoechst 33342 was used to stain the nuclei. All samples were observed under an inverted fluorescence microscope.

### 2.9. Measurement of Cellular ROS

To detect cellular ROS, a DCFH-DA probe (Beyotime Institute of Biotechnology, Shanghai, China) was used according to the manufacturer’s protocol.

### 2.10. Cell Transfection

siRNA targeting SIRT1 (si-SIRT1), agomirs, antagomirs and negative controls (Table 1) were transfected using Lipofectamine 3000 (Invitrogen, Carlsbad, CA, USA). The concentration of agomirs and antagomirs is 50 pmol/mL. All the sequences were from GenePharma (Shanghai, China).

### 2.11. Western Blot

Protein samples were separated by SDS–PAGE. Specific antibodies and relative secondary antibodies were added and incubated. The protein bands were visualized by an enhanced chemiluminescence kit and then quantified by determining the intensity values.

### 2.12. Quantitative RT–PCR

TRIzol reagent (TaKaRa, Dalian, China) was used to isolate total RNA. The TaqMan miRNA Reverse Transcription Kit and TaqMan miRNA assay Kit (GenePharma, Shanghai, China) were used for reverse transcription and real-time PCR, respectively. miRNA expression was normalized to U6.

### 2.13. Luciferase Reporter Assay

The wild-type or mutant 3′UTR of SIRT1 containing miR-485-3p binding sites (GenePharma, Shanghai, China) and ago-485-3p were cotransfected into AML12 cells, followed by Sal A treatment. Then, a Dual-Luciferase Reporter Assay Kit (TransGen, Beijing, China) was used according to the manufacturer’s instructions.

### 2.14. Statistical Analysis

GraphPad Prism 5.0 (La Jolla, CA, USA) was used for statistical analyses. The data were analyzed by one-way analysis of variance, and the results are presented as the mean ± S.D. *p* < 0.05 was considered significant.

## 3. Results

### 3.1. Sal A Ameliorates APAP-Induced Liver Injury

**Figure 1:**
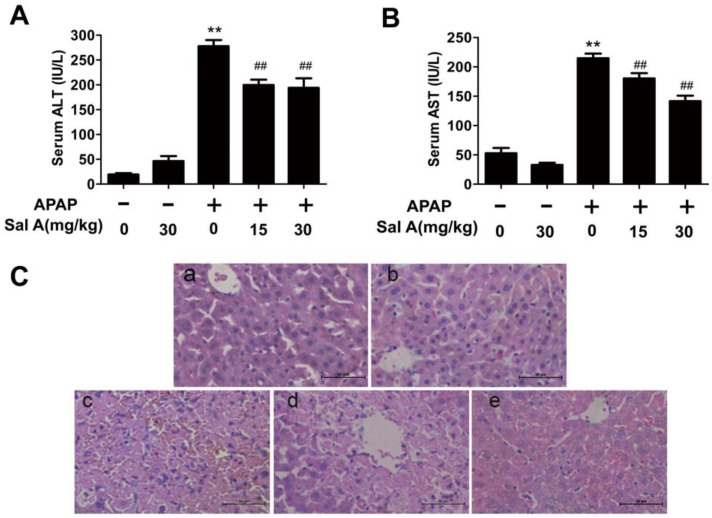
Sal A attenuates liver injury induced by APAP in mice. (**A**,**B**) Serum levels of ALT and AST, *n* = 8. (**C**) H&E staining (200× magnification): a. control; b. Sal A (30 mg/kg); c. APAP (300 mg/kg); d. APAP+Sal A (15 mg/kg); e. APAP+Sal A (30 mg/kg). ** *p* < 0.01 vs. control group, ^##^ *p* < 0.01 vs. APAP group.

AST and ALT are sensitive markers of acute liver injury. The ALT and AST activities were increased by APAP. However, Sal A treatment had a protective effect by significantly eliminating the increases in ALT and AST levels (Figure 1A,B). Consistently, H&E staining revealed severe hepatocyte injury induced by APAP, as evidenced by intrahepatic hemorrhage, lymphocyte infiltration and destruction of the liver structure, which was ameliorated in the Sal A pretreatment group (Figure 1C). Thus, Sal A is effective in protecting against APAP toxicity.

### 3.2. Sal A Ameliorates Hepatic Oxidative Stress In Vivo and In Vitro

**Figure 2:**
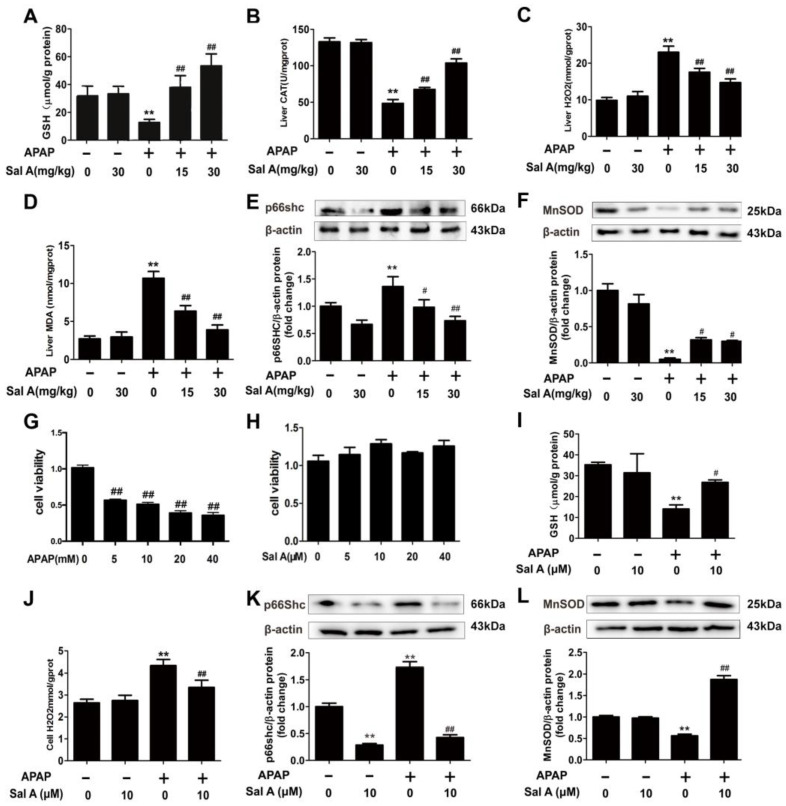
Sal A ameliorates hepatic oxidative stress in vivo and in vitro. (**A**–**F**) Sal A pretreatment was performed for 3 days followed by APAP stimulation in mice. (**A**–**D**) GSH, CAT, H_2_O_2_ and MDA levels in mice, *n* = 8. (**E**,**F**) p66Shc and MnSOD protein expression in mice, *n* = 3. (**G**) AML12 cells were exposed to different concentrations of APAP (0, 5, 10, 20 and 40 mM) for 24 h. Cell viability, *n* = 8. (**H**) AML12 cells were exposed to Sal A for 24 h at different concentrations of 0, 5, 10, 20 and 40 µM. Cell viability, *n* = 8. (**I**–**L**) AML12 cells were incubated with Sal A before exposure to APAP. (**I**,**J**) GSH and H_2_O_2_ levels in AML12 cells, *n* = 8. (**K**,**L**) p66Shc and MnSOD protein expression in AML12 cells, *n* = 3. ** *p* < 0.01 vs. control. ^#^ *p* < 0.05, ^##^ *p* < 0.01 vs. APAP.

Oxidative stress is essential for hepatocellular death in APAP-induced liver injury 5,16]. To determine whether Sal A could protect against oxidative stress induced by APAP, we examined several oxidative stress-related readouts. GSH is one of the most important antioxidants and is sharply decreased by APAP toxicity [19]. Sal A pretreatment ameliorated the decrease in GSH in the APAP-treated group (Figure 2A). Other common indicators of ROS, including H_2_O_2_ and MDA upregulation and CAT downregulation, were detected in the APAP group (Figure 2B–D). However, Sal A pretreatment dose-dependently relieved ROS-induced damage, as indicated by the recovery of these parameters.

P66Shc is expressed ubiquitously in vertebrates. It contributes to intracellular ROS concentrations through plasma membrane oxidase activation and ROS scavenging inhibition [20,21]. MnSOD, another critical ROS mediator located in mitochondria, can prevent peroxynitrite formation [22]. The amelioration of oxidative stress levels by Sal A can also be seen in the protein levels of p66Shc and MnSOD (Figure 2E,F). These results indicate that Sal A treatment protected against APAP-induced oxidative stress.

To evaluate whether Sal A could ameliorate APAP hepatotoxicity in vitro, a cell model was established. According to the cell viability test, 5 mM APAP significantly induced cell death (Figure 2G). Furthermore, 10 µM Sal A resulted in the highest cell viability among the groups (Figure 2H). After administering different treatments to each group, the GSH, H_2_O_2_, p66Shc and MnSOD levels in AML12 cells were measured and were consistent with the in vivo results (Figure 2I–L).

These findings indicate that Sal A could ameliorate APAP-induced oxidative stress in vivo and in vitro.

### 3.3. Sal A Ameliorates Inflammation In Vivo and In Vitro

**Figure 3:**
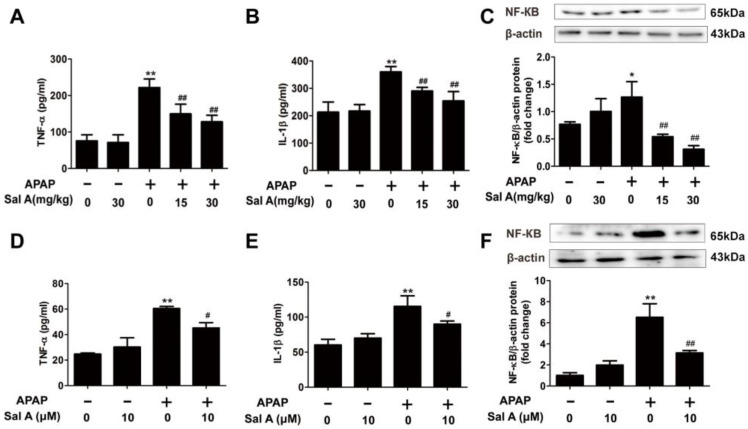
Sal A ameliorates inflammation in vivo and in vitro. (**A**–**C**) Sal A pretreatment for 3 days followed by APAP stimulation in mice. (**A**,**B**) Serum TNF-α and IL-1β levels were determined by ELISA, *n* = 8. (**C**) The protein level of NF-κB in mice, *n* = 3. (**D**–**F**) AML12 cells were incubated with Sal A before exposure to APAP. (**D**,**E**) TNF-α and IL-1β levels were determined by ELISA, *n* = 8. (**F**) The protein levels of NF-κB in AML12 cells, *n* = 3. * *p* < 0.05, ** *p* < 0.01 vs. control. ^#^ *p* < 0.05, ^##^ *p* < 0.01 vs. APAP.

Inflammation is also closely related to APAP hepatotoxicity, and the damage-associated molecular patterns (DAMPs) released from necrotic cells activate the innate immune system and inflammation [23,24]. TNF-α and IL-1β levels were sharply increased after APAP treatment, and Sal A treatment abrogated these increases (Figure 3A,B). NF-κB, which is a key mediator of proinflammatory signaling [25], was also increased after APAP treatment (Figure 3C). However, Sal A pretreatment prevented the increase in NF-κB protein levels. These observations suggest that Sal A can ameliorate APAP-induced inflammation in mice.

Consistently, TNF-α, IL-1β and NF-κB levels were significantly elevated in APAP-treated AML12 cells. However, pretreatment with Sal A notably ameliorated the increase in these inflammatory parameters (Figure 3D–F). Taken together, Sal A ameliorated inflammation in APAP-induced liver injury.

### 3.4. Sal A-Mediated Protection against APAP Involves SIRT1 Activation

**Figure 4:**
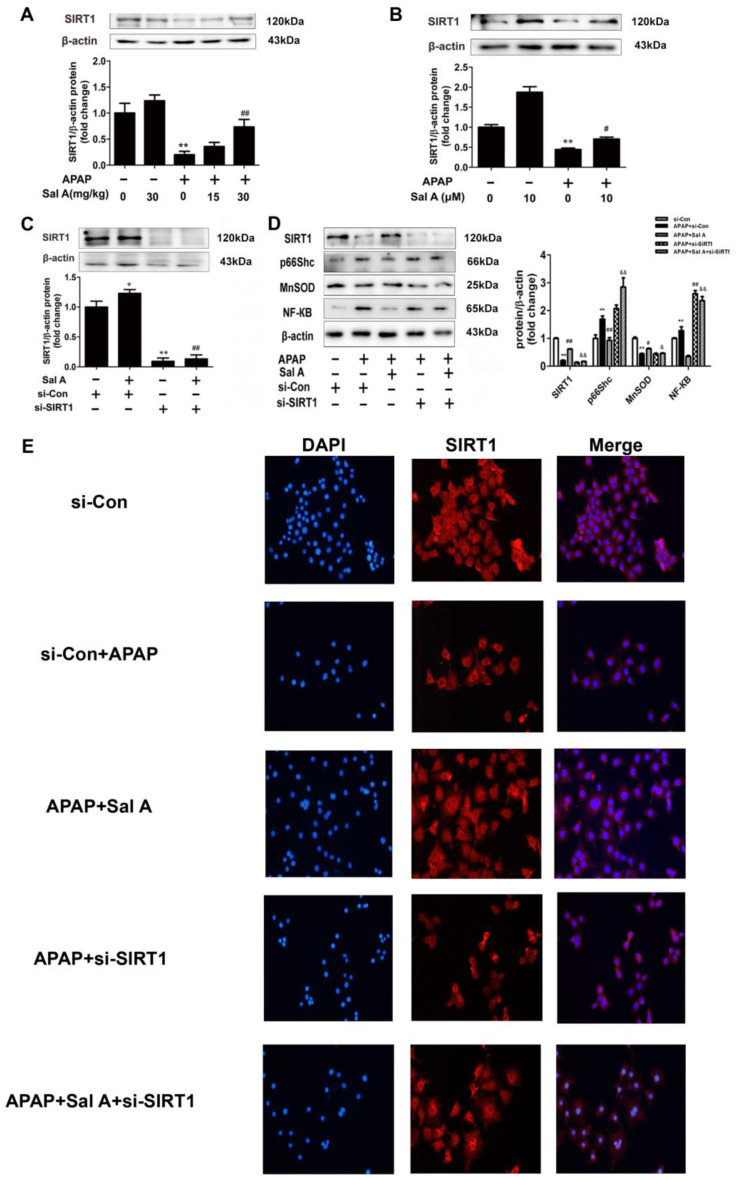
Sal A-mediated protection against APAP involves SIRT1 activation. (**A**) Sal A pretreatment was performed for 3 days followed by APAP stimulation in mice. SIRT1 protein expression in the liver, *n* = 3. (**B**) AML12 cells were incubated with Sal A before exposure to APAP. SIRT1 protein expression in AML12 cells, *n* = 3. (**C**) AML12 cells were transfected with control siRNA or SIRT1 siRNA for 24 h and then incubated with Sal A (10 μM) for 6 h. SIRT1 expression in AML12 cells, *n* = 3. (**D**) AML12 cells were transfected with SIRT1 siRNA or negative control and then incubated with Sal A and APAP. SIRT1, p66Shc, MnSOD and NF-κB protein expressions in AML12 cells, *n* = 3. (**E**) Immunofluorescence analysis of SIRT1 (100× magnification). * *p* < 0.05, ** *p* < 0.01 vs. control or si-control. ^#^ *p* < 0.05, ^##^ *p* < 0.01 vs. APAP or si-con+Sal A. ^&^ *p* < 0.05, ^&&^ *p* < 0.01 vs. APAP+Sal A.

SIRT1 has been reported to rescue oxidative stress and inflammation in several pathologies, including APAP-induced liver injury [4]. Sal A and resveratrol are polyphenolic acids with similar polyphenol structures. Resveratrol has been widely studied as an activator of SIRT1 [26]. We then explored whether SIRT1 was associated with Sal A-mediated protection against APAP-induced liver injury. Hepatic SIRT1 expression was markedly reduced in model mice, and Sal A reversed this loss of SIRT1 (Figure 4A). Consistent with the above data, the protein levels of SIRT1 were decreased in APAP-treated AML12 cells, while pretreatment with Sal A completely abrogated this decrease (Figure 4B). To further explore the potential role of SIRT1 in Sal A-mediated protection against APAP-induced hepatotoxicity, siRNA-mediated knockdown of SIRT1 was used in AML12 cells. Compared to control siRNA, SIRT1 expression was nearly blocked by SIRT1 siRNA, and Sal A did not promote SIRT1 (Figure 4C). In addition, after the transfection of si-SIRT1, the efficacy of Sal A was diminished, as indicated by the increased protein levels of p66Shc and NF-κB and the decrease in MnSOD (Figure 4D). Immunofluorescence was used to more directly investigate whether the protective effect of Sal A involves SIRT1 activation. SIRT1, which was marked with red fluorescence, was sharply decreased by APAP treatment and recovered when the cells were pretreated with Sal A. However, the transfection of si-SIRT1 almost diminished Sal A-mediated protection, as indicated by low levels of red fluorescence (Figure 4E). These findings suggest that Sal A-mediated protection against APAP toxicity involves SIRT1 activation.

### 3.5. Selection of SIRT1-Targeting miRNAs in APAP-Induced Liver Injury

**Figure 5:**
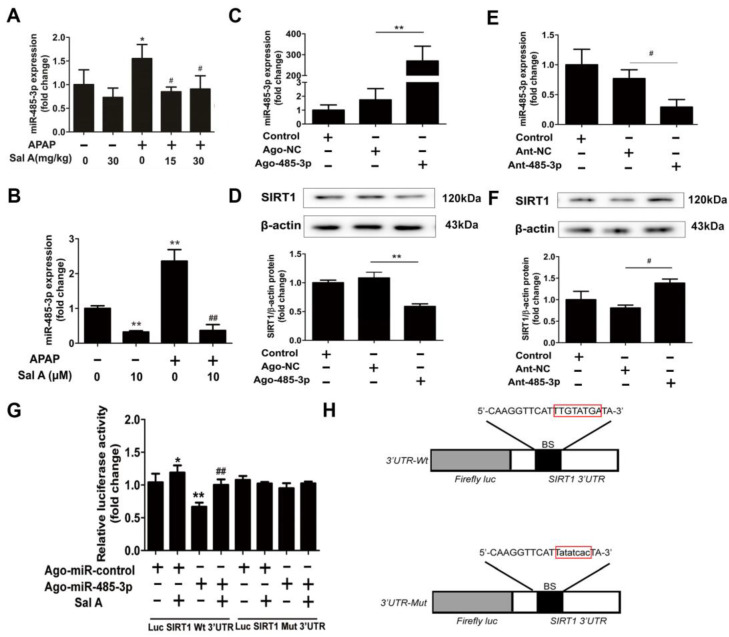
miR-485-3p regulates SIRT1 expression in AML12 cells. (**A**) Sal A pretreatment was performed for 3 days followed by APAP stimulation in mice. miR-485-3p expression in mice, *n* = 3. (**B**) AML12 cells were incubated with Sal A before exposure to APAP. miR-485-3p expression in AML12 cells, *n* = 3. (**C**–**F**) Ago-485-3p, ant-485-3p or negative control was transfected into AML12 cells. (**C**,**E**) miR-485-3p expression, *n* = 3. (**D**,**F**) SIRT1 expression, *n* = 3. (**G**) Dual luciferase assay. Luciferase constructs containing wild-type or mutant SIRT1-3′-UTR and ago-485-3p or negative control were cotransfected into AML12 cells followed by Sal A treatment, *n* = 3. (**H**) Luciferase constructs with 3′UTR-wt or 3′UTR-mut of SIRT1. BS, binding site. * *p* < 0.05, ** *p* < 0.01 vs. control, ago-NC or ant-NC. ^#^ *p* < 0.05, ^##^ *p* < 0.01 vs. APAP.

miRNAs, which are small noncoding RNAs, can negatively regulate the expression of mRNAs and proteins [11]. According to a literature review, SIRT1 can be regulated by miRNAs in other models [27]. This theoretical basis and our preliminary experiment prompted us to evaluate the role of microRNAs in the regulation of SIRT1 during APAP toxicity. Thus, GEO datasets and the literature were screened to identify miRNAs that were upregulated in the liver after APAP treatment. It was reported that 33 different miRNAs were increased by APAP toxicity [28]. However, when TargetScan was used, seven miRNAs were predicted to target SIRT1 (Table 2). Among these miRNAs, only miR-485-3p was conserved between humans and mice (Table 3). To determine whether the expression of this miRNA was in accordance with our hypothesis, miR-485-3p expression was measured by qRT–PCR in vivo and in vitro. In vivo, miR-485-3p expression was significantly elevated in the APAP group, and Sal A pretreatment ameliorated this increase (Figure 5A). To our surprise, miR-485-3p expression showed the same trend and was even more obvious in vitro (Figure 5B).

### 3.6. miR-485-3p Regulates SIRT1 Expression In Vitro

After observing the contrasting changes in miR-485-3p and SIRT1 expression, a miR-485-3p agomir (ago-485-3p) or antagomir (ant-485-3p) was transfected into AML12 cells to confirm whether miR-485-3p influences the expression of SIRT1. miR-485-3p expression was dramatically increased by ago-485-3p and significantly downregulated by ant-485-3p. Ago-485-3p downregulated SIRT1 protein levels, while ant-485-3p upregulated SIRT1 protein levels (Figure 5C–F).

To assess whether miR-485-3p could target the SIRT1 3′-UTR, the wild-type or mutated SIRT1 3′UTR luciferase construct was transfected into AML12 cells that were exposed to Sal A. The luciferase activity of wild-type SIRT1 3′-UTR was partly suppressed by ago-485-3p. Compared to the ago-miR-485-3p group, Sal A pretreatment increased luciferase activity. Nevertheless, there was no repression that was shown for the mutated SIRT1 3′ UTR (Figure 5G,H). The results revealed two conclusions: (1) miR-485-3p regulates SIRT1 expression in AML12 cells; (2) the increase in SIRT1 by Sal A occurs at least partly through miR-485-3p regulation.

### 3.7. Sal A Alleviates APAP-Induced Liver Injury though the miR-485-3p/SIRT1 Pathway

**Figure 6:**
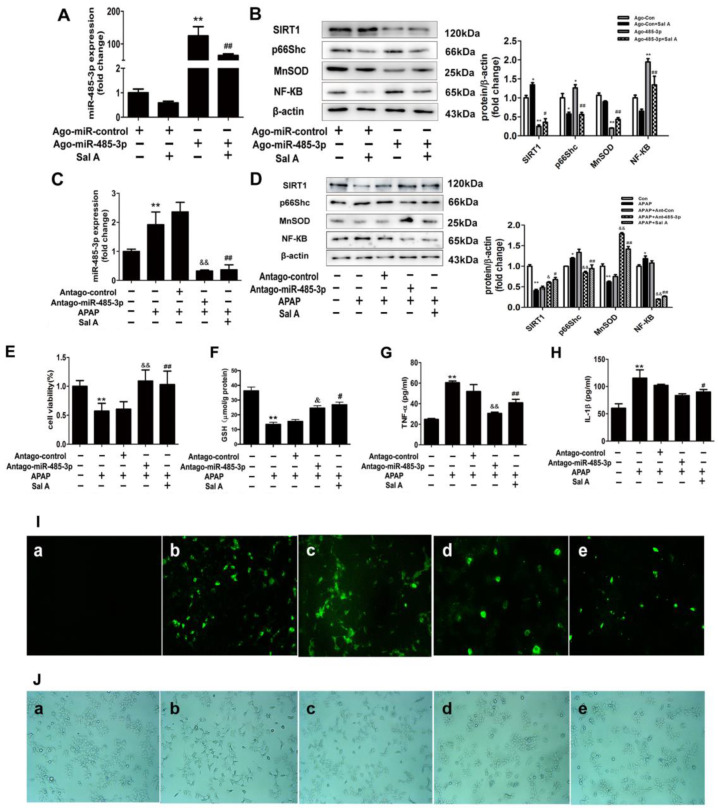
Sal A alleviates APAP-induced liver injury by controlling the miR-485-3p/SIRT1 pathway. (**A**,**B**) Ago-485-3p or negative control was transfected into AML12 cells followed by Sal A treatment. (**A**) miR-485-3p expression, *n* = 3. (**B**) SIRT1, p66Shc, MnSOD and NF-κB expression in AML12 cells, *n* = 3. (**C**–**I**) Antago-485-3p or the negative control was transfected into AML12 cells followed by Sal A treatment and APAP administration. (**C**) miR-485-3p expression, *n* = 3. (**D**) SIRT1, p66Shc, MnSOD and NF-κB expression in AML12 cells, *n* = 3. (**E**) Cell viability, *n* = 8. (**F**) GSH level in AML12 cells, *n* = 8. (**G**,**H**) TNF-α and IL-1β levels in the culture medium were determined by ELISA, *n* = 8. (**I**) Intracellular ROS levels, *n* = 3. Intracellular ROS levels (green) in AML12 cells were determined by the fluorescent probe DCFH-DA, and (**J**) cellular morphology and structure were determined by bright image (100× magnification), *n* = 3: a. antago-control; b. APAP; c. antago-control+APAP; d. antago-485-3p+APAP; e. APAP+Sal A. * *p* < 0.05, ** *p* < 0.01 vs. ago-control or antago-control; ^#^ *p* < 0.05, ^##^ *p* < 0.01 vs. APAP or ago-485-3p, ^&^ *p* < 0.05, ^&&^ *p* < 0.01 vs. APAP+ant-NC.

After determining the relationship between Sal A, miR-485-3p and SIRT1, we were interested in determining the role of this pathway. First, ago-485-3p was transfected into AML12 cells, and the high expression of miR-485-3p induced by agomiR transfection was sharply decreased by Sal A pretreatment (Figure 6A). This finding indicates that Sal A has an influence on miR-485-3p expression under normal conditions. Furthermore, the expression levels of critical proteins in the miR-485-3p-SIRT1 pathway were tested. The transfection of ago-485-3p downregulated SIRT1 and MnSOD and upregulated p66Shc and NF-κB compared to the control group, and pretreatment with Sal A reversed these effects (Figure 6B). These findings indicate that Sal A can regulate the function of the miR-485-3p/SIRT1 pathway under normal conditions.

Under pathological conditions, the relationship between Sal A and the miR-485-3p/SIRT1 pathway was examined. The transfection of ant-485-3p downregulated the expression of miR-485-3p compared with the APAP group, which was the same effect as Sal A (Figure 6C). Similarly, SIRT1 and MnSOD were obviously upregulated, and p66Shc was downregulated after ant-485-3p transfection compared to the APAP group (Figure 6D). Moreover, the transfection of ant-485-3p increased cell viability and GSH level, and helped maintain normal cell morphology after APAP treatment (Figure 6E,F,J). Inflammatory indicators, including NF-κB, TNF-α and IL-1β, were ameliorated after ant-485-3p pretreatment (Figure 6D,G,H). ROS accumulation, which was represented by green fluorescence, decreased after pretreatment with ant-485-3p (Figure 6I). These findings indicate that (1) inhibition of the miR-485-3p/SIRT1 pathway protects AML12 cells from APAP-induced oxidative stress and inflammation and (2) Sal A has an influence on the miR-485-3p/SIRT1 pathway.

## 4. Discussion

APAP is widely used as an analgesic. It is notable that APAP overdose could result in severe liver injury and even acute liver failure [1,3,4]. Except for the medication N-acetylcysteine (NAC), for APAP overdose patients, effective treatment methods remain lacking [29]. This study demonstrated that (1) inhibition of the miR-485-3p/SIRT1 pathway protects against oxidative stress and inflammation induced by APAP and (2) Sal A alleviates APAP-induced liver injury through the miR-485-3p/SIRT1 pathway.

SIRT1 protects against APAP-induced liver injury, liver fibrosis and other hepatic diseases [4,30,31,32]. In this study, SIRT1 expression was detected in both mice and AML12 cells. Compared to the control group, lower SIRT1 protein expression was found in APAP-treated mice and AML12 cells exposed to APAP for 24 h. SIRT1 has been reported to alleviate oxidative stress and inflammation by inhibiting p66shc and regulating NF-κB [24,27]. In the current study, p66shc and NF-κB protein levels were increased by APAP stimulation compared to the control group. Oxidative damage and inflammation induced by APAP were further exacerbated by SIRT1 inhibition in AML12 cells. Similar to previous studies, these findings indicate that SIRT1 inhibits oxidative stress and inflammation in APAP-induced liver injury. Thus, we were interested in finding a molecule that could reverse the downregulation of SIRT1 during APAP-induced toxicity.

miRNAs, which are small, noncoding RNAs, play fundamental roles in gene regulation [11]. Several SIRT1-targeted miRNAs have been identified in stress conditions. In a previous study, miR-34a was shown to suppress SIRT1 in nonalcoholic fatty liver disease [27]. However, in APAP-related studies, most researchers focused on biomarkers in the early stage of APAP toxicity, such as miR-122 [33,34]. Thus, there is great potential to investigate the effect of miRNAs on APAP toxicity. The expression of miR-485-3p, which could target the SIRT1 3′-UTR, was upregulated in APAP-induced liver injury, which was consistent with our prediction. Furthermore, after the transfection of ago-485-3p in AML12 cells, SIRT1 protein level was decreased compared to the control, and SIRT1 protein expression was increased in response to ant-485-3p. Luciferase assays demonstrated that miR-485-3p could directly bind to the SIRT1 3′-UTR. Overall, miR-485-3p plays a crucial role in the regulation of SIRT1 expression.

miR-485-3p has been shown to be effective in the progression of several diseases. It has been demonstrated that miR-485-3p can target PGC-1α to suppress breast cancer cell metastasis. On the other hand, miR-485-3p affects cellular iron homeostasis by binding to ferroportin [35]. However, no article has focused on the relationship between miR-485-3p and APAP. In this study, the inhibition of miR-485-3p relieved cell death, ROS generation and inflammation induced by APAP. In addition, miR-485-3p silencing relieved APAP-induced SIRT1 suppression, implying that SIRT1 is involved in the protective effects of miR-485-3p.

Notably, polyphenols are beneficial against many diseases by targeting miRNAs. Sal A, a phenolic compound extracted from *Salvia miltiorrhiza* Bunge, is an effective anti-inflammatory and antioxidant agent [12,13]. In this study, Sal A was found to reduce ALT and AST activities induced by APAP in mice. Furthermore, the protective effect of Sal A was confirmed by liver histological evaluation. However, Sal A did not completely reduce the elevation of ALT and AST caused by APAP. The possible reason for this is that the damage degree caused by APAP is more severe than other liver injuries [36,37]. Similarly, for this reason, some studies were conducted using prophylactic treatment [38,39], as in our paper. Mechanistically, pretreatment with Sal A was associated with miR-485-3p downregulation and SIRT1 upregulation. These findings suggested that Sal A-mediated protection, at least partly, occurred through the miR-485-3p/SIRT1 pathway.

**Figure 7:**
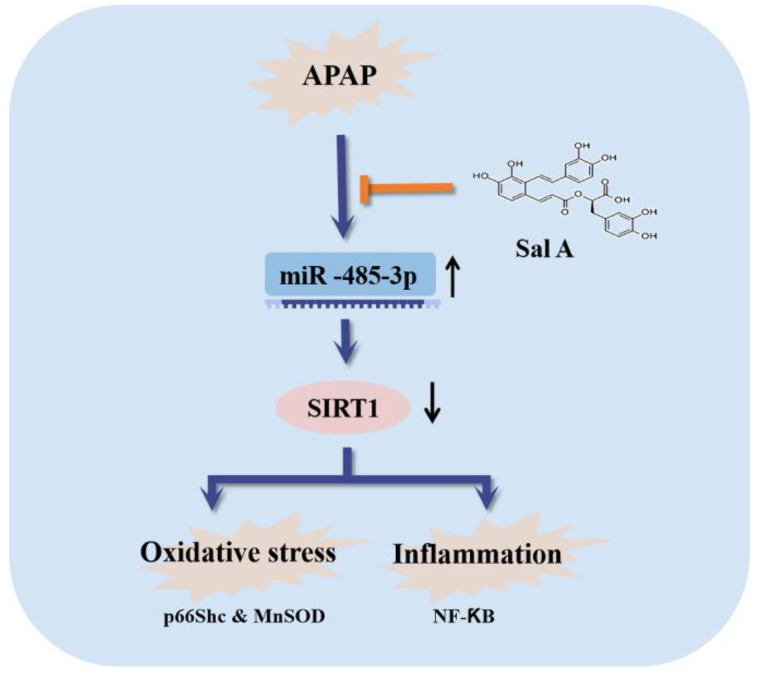
Sal A attenuates APAP-induced oxidative stress and inflammation by repressing miR-485-3p-mediated inhibition of SIRT1.

The occurrence of APAP-induced liver injury is closely related to the depletion of GSH in the liver [5]. In our study, we found that Sal A pretreatment and miR-485-3p silencing could significantly improve the decline in GSH levels caused by APAP in vivo and in vitro. In addition, the DAMPs released from necrotic cells, which are caused by APAP toxicity, activate the innate immune system and abrogate the increase in TNF-α and IL-1β [4,6,23]. In the present study, the oxidative stress and inflammation caused by APAP toxicity were reversed by Sal A pretreatment in vivo and in vitro. Moreover, the induction of miR-485-3p was reversed by Sal A in AML12 cells, and the inhibition of the miR-485-3p/SIRT1 pathway had similar effects as Sal A treatment, suggesting that Sal A has the potential to reduce APAP toxicity by inhibiting the miR-485-3p/SIRT1 pathway (Figure 7).

Although hepatocellular injury due to oxidative stress and inflammation is considered a hallmark of APAP overdose toxicity, APAP overdose also induces damage in the brain. It is not completely clear whether brain injury from APAP toxicity was attributed to secondary effects of the liver failure or was induced by the direct effect of APAP and its toxic metabolites in the brain [40]. A recent study showed that APAP can pass the blood–brain barrier (BBB) and be taken up by the dopaminergic transport system into the substantia nigra (SN), which is a potential risk factor for the development of PD [2]. Paradoxically, low doses of APAP have been reported to produce the opposite, neuroprotective effects [41]. Therefore, more experiments are needed to further demonstrate the mechanisms and the pathways mediating APAP toxicity in PD in vitro, in animal models and most importantly in humans. A better understanding of APAP mechanism in the brain may provide beneficial therapeutic approaches for neurodegenerative diseases. 

## 5. Conclusions

Collectively, these results demonstrated that the miR-485-3p/SIRT1 signaling pathway may function as an attractive pharmacological target against liver injury induced by APAP through inhibition of oxidative stress and inflammation. Meanwhile, Sal A may be a new potential candidate for the treatment of APAP toxicity.

## Figures and Tables

**Table 1:** Sequences of agomirs, antagomirs, mRNA and siRNAs.

Name	Sequences (5′-3′)	Accession Number
agomir targeting miR-485-3p	AGUCAUACACGGCUCUCCUCUC GAGGAGAGCCGUGUAUGACUUU	MIMAT0003129
agomir negative control	UUCUCCGAACGUGUCACGUTT ACGUGACACGUUCGGAGAATT	MIMAT0000295
antagomir targeting miR-485-3p	GAGAGGAGAGCCGUGUAUGACU	MIMAT0003129
antagomir negative control	CAGUACUUUUGUGUAGUACAA	MIMAT0000295
siRNA targeting SIRT1	CCCUGUAAAGCUUUCAGAA (TT) UUCUGAAAGCUUUACAGGG (TT)	NM_019812.3
siRNA negative control	ACGUGACACGUUCGGAGAA (TT) UUCUCCGAACGUGUCACGU (TT)	M403861200
SIRT1 mRNA--F	CCCAGCTCCAGTCAGAACTAT	NM_019812.3
SIRT1 mRNA--R	TTGGCACCGATCCTCGAAC	
β-actin mRNA--F	TTCGTTGCCGGTCCACACCC	NM_001101.5
β-actin mRNA--R	GCTTTGCACATGCCGGAGCC	

**Table 2:** Selection of putative SIRT1-targeting miRNAs in the liver after APAP overdose.

miRNA Name				
Increased miRNA	mmu-miR-297a	mmu-miR-483	mmu-let-7d *	mghv-miR-M1-2
	mmu-miR-574-3p	mmu-miR-709	mmu-miR-466 g	mmu-miR-466 h
	mmu-miR-466f-3p	mmu-miR-1224	mmu-miR-574-5p	mmu-miR-467a *
	mmu-miR-671-5p	mmu-miR-467b *	mmu-miR-207	mmu-miR-669c
	mmu-miR-483 *	mmu-miR-877 *	mmu-miR-467e *	mmu-miR-468
	mmu-miR-297b-3p	mmu-miR-197	mmu-miR-672	mmu-miR-328
	mmu-miR-466c-5p	mmu-miR-485 *	mmu-miR-689	mmu-miR-188-5p
	mmu-miR-669a	mmu-miR-721	mmu-miR-710	mmu-miR-711
	mmu-miR-466d-3p			
SIRT1-targeted miRNAs	miR-483	miR-467b *	miR-467e *	miR-297b-3p
	miR-485 *	miR-466d-3p	miR-672	
Conservation	miR-485 *			

(* indicates that the mature species of miRNA were found at low levels from the opposite arm of a hairpin).

**Table 3:** The predicted binding region between SIRT1 and miR-485-3p.

	Predicted Consequential Pairing of Target Region (Top) and miRNA (Bottom)	Accession Number
Position 232-238 of SIRT1 3′ UTR	5′…CUUUCAAGGUUCAUUUGUAUGAU…	NM_019812.3
	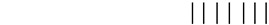	
mmu-miR-485-3p	3′ UCUCUCCUCUCGGCACAUACUG	MIMAT0003129
Position 280-286 of SIRT1 3′ UTR	5′…UUUUAAAGGUUCAUUUGUAUGAU…	NM_012238.5
	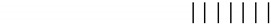	
hsa-miR-485-3p	3′ UCUCUCCUCUCGGCACAUACUG	MIMAT0002176

## Data Availability

All the data are available within the article.
